# Current overview of *S*-nitrosoglutathione (GSNO) in higher plants

**DOI:** 10.3389/fpls.2013.00126

**Published:** 2013-05-08

**Authors:** Francisco J. Corpas, Juan D. Alché, Juan B. Barroso

**Affiliations:** ^1^Departamento de Bioquímica, Biología Celular y Molecular de Plantas, Estación Experimental del Zaidín, Consejo Superior de Investigaciones CientíficasGranada, Spain; ^2^Grupo de Señalización Molecular y Sistemas Antioxidantes en Plantas, Unidad Asociada al Consejo Superior de Investigaciones Científicas (EEZ), Área de Bioquímica y Biología Molecular, Universidad de JaénJaén, Spain

## Introduction

**Figure 1:**
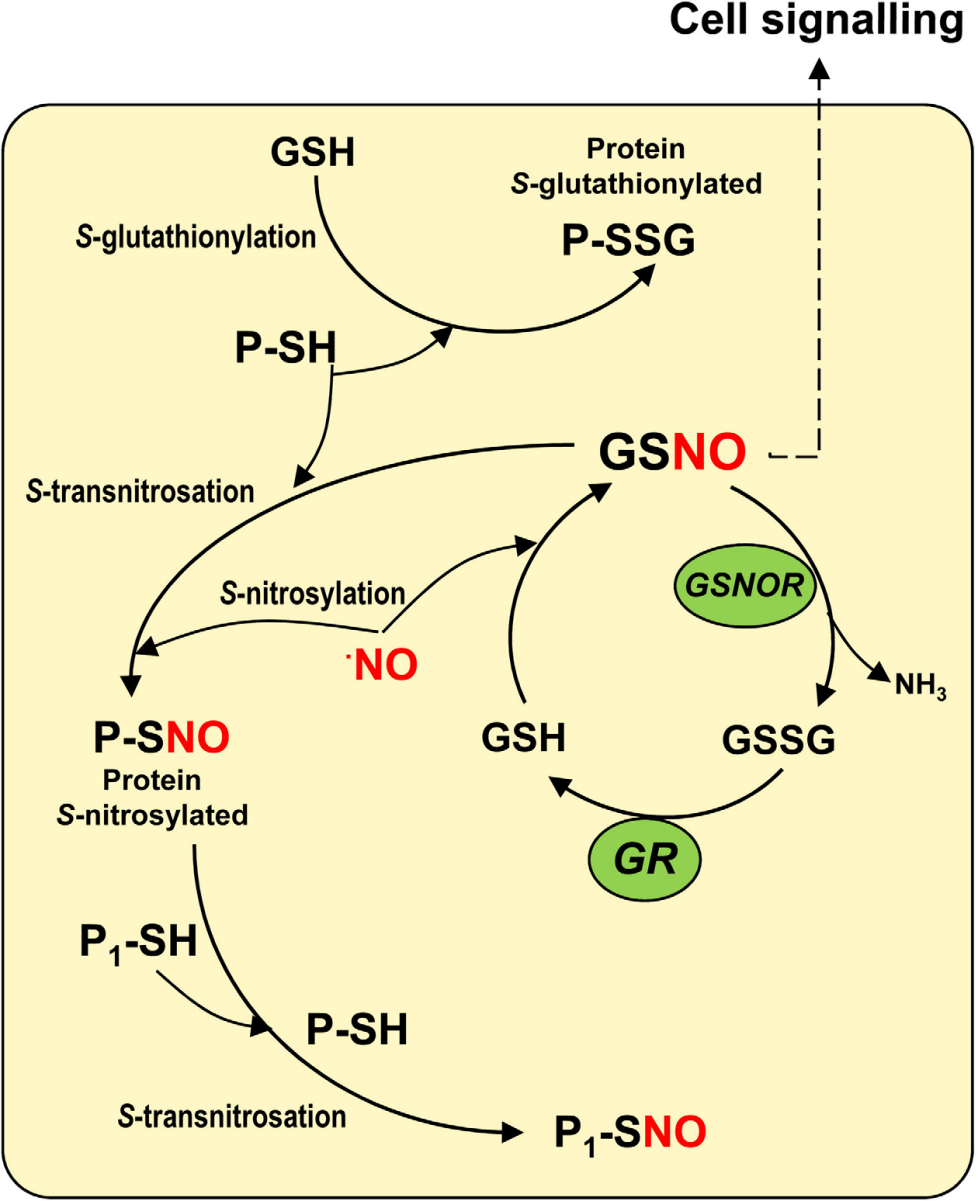
**Model of the *S*-nitroglutathione (GSNO) metabolism in cells.** The interaction between reduced glutathione (GSH) and nitric oxide (NO) enables GSNO to be generated by a process of *S*-nitrosylation. GSNO could be decomposed by the GSNO reductase to oxidized glutathione (GSSG) which is the substrate of the glutathione reductase (GR) that regenerates the reduced glutathione. GSNO, regarded as the most abundant low-molecular mass (LMM) *S*-nitrosothiol, can interact with specific sulfhydryl (-SH) groups of proteins to produce high-molecular mass (HMM) *S*-nitrosothiols in a process called *S*-transnitrosation (Hogg, 2002). HMM S-nitrosothiols can also transfer NO to the sulfhydryl (-SH) groups of other proteins (P_1_-SH) through a process of *S*-transnitrosation between proteins. On the other hand, GSH can interact with specific sulfhydryl (-SH) groups of proteins in a process known as *S*-glutathionylation.

*S*-nitrosoglutathione is a nitric oxide-derived molecule, generated by the interaction of nitric oxide (NO) with reduced glutathione (GSH) in a process called *S*-nitrosylation (Figure 1). The reaction appears to take place either through the formation of N_2_O_3_ or the addition of NO to a glutathionyl radical formed during this reaction (Broniowska et al., 2013). GSNO is regarded as an intracellular NO reservoir as well as a vehicle of NO throughout the cell, which enables NO biological activity to expand. GSNO is also considered to be the most abundant low-molecular-mass (LMM) *S*-nitrosothiol (SNO). This family includes other molecules such as *S*-nitrosocysteine (CySNO) and *S*-nitrosocysteinylglycine (GlyCySNO), which have been the subject of less study in the field of plant research. There is another group of SNOs called high-molecular mass (HMM) SNOs which are produced by NO binding to sulfhydryl (-SH) groups present in specific cysteine residues of proteins. Figure 1 shows a simple model of GSNO metabolism and its interactions with other molecules in cells where different reactions including *S*-nitrosylation, *S*-transnitrosation, and *S*-glutathionylation are involved (Hogg, 2002; Martínez-Ruiz and Lamas, 2007). In plants, research has focused on the importance of total SNOs in specific stress situations (Feechan et al., 2005; Chaki et al., 2011a) and on the identification of the potential protein targets of *S*-nitrosylation as this kind of post-translational modification can alter the function of the affected proteins (Astier et al., 2012). Initial studies in this area exogenously applied GSNO in order to identify the pool of potential protein candidates (Lindermayr et al., 2005). However, less attention has been paid to the abundance, distribution, and modulation of endogenous GSNO under natural and stress conditions. In this article, we will provide a current overview of GSNO in higher plants.

## Detection and quantification of GSNO in plants

Although, a considerable number of studies of NO in plants are available, much less information exists regarding SNOs and, more specifically, GSNO. This is mainly explained by the fact that the determination of GSNO in plant samples still presents a challenge in analytical terms due to several technical obstacles and the often lengthy sample preparation procedures required. In addition, other potential problems are caused by the intrinsic instability of GSNO in plant samples. Thus, the determination of GSNO can be affected by light, metal-catalyzed GSNO decomposition, enzymatic degradation as a result of endogenous GSNO reductase activity and a reduction in the *S*-NO bond caused by reductants and endogenous thiols.

In higher plants, two different app-roaches to detect GSNO have been reported: immunohistochemical analysis using commercial antibodies against GSNO (Barroso et al., 2006; Valderrama et al., 2007) and liquid chromatography-electrospray/mass spectrometry (LC-ES/MS) (Airaki et al., 2011). These techniques have provided some initial background data on cell localization in different organs and on GSNO content under development and adverse stress conditions. Whereas immunohistochemical localization using fluorescence probe as secondary antibody can provide localization a relative abundance with high sensitivity, LC-ES/MS is the technique that provides a most consistent quantification. The reported GSNO content ranges between 3 and 8 nmol GSNO g^−1^ fresh weight (Airaki et al., 2011) which is in the same range of oxidized glutathione (GSSG).

## Function of GSNO under adverse environmental conditions

At present, some data shows that GSNO is an important molecule in the mechanisms of response to biotic and abiotic stress. Immunohistochemical analysis using confocal laser scanning microscope (CLSM) in several plant species under different stress conditions has enabled the spatial and relative content of GSNO to be determined. In pea plants, the content of GSNO localized in leaf collenchyma cells and under 50 μM cadmium stress was drastically reduced, which was accompanied by a 31% reduction in GSNOR activity (Barroso et al., 2006). In addition, *Arabidopsis thaliana* exposed to a toxic concentration of arsenic causing nitro-oxidative stress showed a significant reduction in GSNO content detected by LC-ES/MS. However, GSNOR activity, which increased significantly, showed an opposite tendency (Leterrier et al., 2012). In the case of olive plants grown in the presence of 200 mM NaCl, the localization and relative GSNO content evaluated by CLSM were totally different, with salinity causing a marked increase in GSNO activity, mainly in the vascular tissue (Valderrama et al., 2007).

In sunflower plants, GSNO has been studied under biotic and abiotic stresses. CLSM analysis of hypocotyl sections of plants exposed to abiotic stress (mechanical wounding and high temperatures) showed a general accumulation of GSNO in all hypocotyl cells, with a concomitant reduction in GSNOR activity, thus mediating nitrosative stress (Chaki et al., 2011a,b). Similar behavior was observed in sunflower under biotic stress, specifically in relation to the fungus *Plasmopara halstedii*. However, it is interesting to note that GSNO was observed to be localized and distributed in the sunflower hypocotyls of the resistant cultivar, while GSNO showed a general and homogenous distribution in all hypocotyl cell types. This appears to contribute to its resistance to fungus, with GSNO after infection being exclusively redistributed to the epidermal cells which are usually this pathogen's penetration sites in sunflowers (Chaki et al., 2009). GSNO mobilization has also been described in wounded *Arabidopsis* leaves where GSNO content increased and showed a uniform distribution pattern, whereas, in systemic leaves, GSNO was first detected in vascular tissues and later extended to the parenchyma cells (Espunya et al., 2012). These findings in relation to different plant species and under different stresses bolster the notion that GSNO appears to be a mobile signal in response to diverse types of stress. Although, the experimental evidence suggests the GSNO movement between plant cells and organs, future specific experiments will be needed to confirm it.

## *S*-nitrosoglutathione and plant development

The effect of NO on seed germination, root architecture, development, and fruit ripening has been routinely studied using NO donors such as sodium nitroprusside. However, more recently, GSNO has begun to be used as it is considered to be a more physiological NO donor (Liu et al., 2007; Zandonadi et al., 2010; Semchuk et al., 2011). However, to our knowledge, there is no information on the content of endogenous GSNO during these plant processes. As mentioned earlier, the use of LC-ES/MS to detect and quantify GSNO has provided some initial data on GSNO content in plant organs. Thus, analysis of GSNO in the main organs of pepper plants have indicated that GSNO was most abundant in roots, followed by leaves and stems, which directly correlated with the content of NO in each organ and inversely correlated with GSNOR activity (Airaki et al., 2011). Very recently, it has been also reported the subcellular localization of GSNO in pea leaves by electron microscopy immunocytochemistry and immunogold particles were clearly visible in cytosol, chloroplasts, mitochondria, and peroxisomes (Barroso et al., 2013).

On the other hand, the involvement of NO in plant reproductive biology has been reported (Bright et al., 2009; Zafra et al., 2010). Thus, NO can act as a negative regulator of pollen tube growth in plants such as *Lilium longiflorum*, *Arabidopsis thaliana*, and *Paulownia tomentosa* (Prado et al., 2004, 2008; He et al., 2007) and as a positive stimulus of pollen tube growth in *Pinus bangeana* in a dose-dependent manner (Wang et al., 2009). Recently, analysis of GSNO by LC-ES/MS in olive pollen subjected to *in vitro* germination has shown the presence of GSNO (unpublished data) whose content closely correlated with the NO content previously reported (Zafra et al., 2010) and inversely correlated with *GSNOR* gene expression.

## Conclusions

The study of GSNO, which is part of the metabolism of NO in higher plants, has begun to increase our knowledge of the physiological significance of this universal molecule that is involved in almost all the process where GSNO has been studied. Consequently, the analysis of GSNO content and metabolism during plant development and under environmental stress conditions presents a new challenge in relation to the signaling properties of GSNO.
